# Practicality of a patient self-assessment checklist to manage dementia risk factors in GP practices

**DOI:** 10.1038/s41598-025-01455-8

**Published:** 2025-05-16

**Authors:** Francisca S. Rodriguez, Hanna L. Knecht, Bernhard Michalowsky, Doreen Goerss, Stefan Teipel, Wolfgang Hoffmann, Marina Boccardi

**Affiliations:** 1 German Center for Neurodegenerative Diseases (DZNE), Ellernholzstr. 1-2, 17489 Greifswald, Germany; 2 German Center for Neurodegenerative Diseases (DZNE), Gehlsheimer Str. 20, 18147 Rostock, Germany; 3 Department of Psychosomatic Medicine and Psychotherapy, University Medicine Rostock, Gehlsheimer Str. 20, 18147 Rostock, Germany; 4 University of Applied Sciences and Arts of Southern Switzerland (SUPSI), via Violino 11, 6928 Manno, Switzerland; 5 German Center for Neurodegenerative Diseases (DZNE), RG Psychosocial Epidemiology & Public Health, Ellernholzstr. 1-2, 17489 Greifswald, Germany

**Keywords:** Dementia, Alzheimer’s, Risk factor, Prevention, Lifestyle, Health care, Risk factors

## Abstract

A screening tool may help determine who in the population is exposed to which dementia risk factor and, in this way, prevent or delay symptoms. With this study, we examined the practically of using a patient self-completion screening checklist for detecting dementia risk factors with general practitioners (GP). The checklist ‘*CogFit’* was developed based on systematic reviews and meta-analyses. Fourteen GPs (average age 44.8 years; 71.4% female) tested it with their patients and reported their experiences in a questionnaire and the System Usability Scale (SUS). In our survey, 57.2% of the GPs considered the checklist useful and most GPs (83%) indicated that the current version is sufficient. The user-friendliness of the *CogFit* checklist was good (SUS score M = 73.13, SD = 10.45). Most GPs (71.4%) reported having gained important information about their patients. 69% gave lifestyle advice and 61.5% referred their patients to brochures or courses. Yet, about two thirds (64.3%) reported an increased workload and four GPs disagreed to use the checklist in future. Our results indicate an overall good practicality of our patient self-completion checklist (*CogFit*) in primary care. Further research involving patients and estimating the overall effectiveness on cognitive health is needed.

## Introduction

Primary prevention is considered the most promising strategy to avert or delay dementia^1^. The World Health Organization and the Lancet Commission identified through intensive literature reviews several risk factors that can reduce the risk of dementia^2^. These could, if all properly addressed, prevent up to 40% of dementia cases^3^. All of these risk factors make great candidates for prevention programs to implement the goal of the World Health Organization’s (WHO) Global Action Plan against dementia^4^.

The first studies testing interventions that address dementia risk factors are already being conducted. The most famous is the *Finnish Geriatric Intervention Study to Prevent Cognitive Impairment and Disability (FINGER)*, a multimodal intervention that has shown to reduce the risk of developing new chronic diseases^5^ and improve cognitive abilities^6^. In the hope of providing a method to prevent dementia, the World-Wide FINGERS Network was founded. It included many international studies that follow the example of the FINGER study^7^, e.g., the *U.S.Pointer* study or the *Singapore SINGER* study. Despite positive results, a Cochrane review of the current state of evidence on such interventions confirmed only small improvement in cognitive function^8^. It is important to note that effects on dementia incidence can only be estimated after a very long observation period. Yet, many intervention studies have just started. Moreover, even with small effects, prevention can be cost-effective, as a systematic review, not restricted to cognitive health, pointed out^9^. Estimates from the UK suggest that dementia prevention interventions, which target smoking, hearing aids, and hypertension, could help save £1.8 billion annually because they are expected to reduce dementia prevalence by more than eight percent^10^. Accordingly, despite initial small effects over a short time period, the long-term effect on large populations can be meaningful. A first step for initiating such interventions is the screening for risk factors in the general population.

While algorithms to estimate a person’s total dementia risk have been developed^11^, such tools do not entail an actionable plan to modify individual risk factors. Moreover, they are usually conceived by specialists so that they might not be practical for the general population. For example, the CogDrisk dementia score contains 90 questions (including non-modifiable risk factors) and takes more than half an hour to complete^11^. Many risk scores include biomarker and imaging data that are not immediately available to everyone^12^. Nonetheless, findings from implementation science suggest that checklists are useful. One example is the ‘frailty index’ consisting of 38 items (e.g., self-rated health, psychiatric and medical conditions, hearing and mobility problems, problems in daily life), which was associated with mortality^13^. Moreover, the interRAI instruments, which comprise several assessments for adults in long-term care settings, community mental health, emergency departments, and for mobile crisis teams, have been successfully implemented. One of these assessments is the ‘interRAI Check-Up Self-Report’ instrument for cognitive, psychosocial, and functional problems of older people. A study has shown that using this checklist in primary health settings can improve wellbeing, positive mood, and social integration^14^. Indeed, the first contact point for many patients in health care systems worldwide are the general practitioners (GP) in primary care. Therefore, primary care offers a key entry point for identifying and addressing risk factors of dementia. Accordingly, a patient self-assessment checklist that specifically screens for modifiable dementia risk factors allows physicians to take preventative actions. In this way, it is more useful than just generating a risk score.

To investigate a possible new approach for dementia prevention among the cognitively still healthy population, the objective of this work was to examine the use of a patient self-assessment checklist with general GPs. Specifically, we investigated GPs’ perspectives on the practicality of a patient self-assessment checklist that screens for modifiable dementia risk and protective factors *(CogFit)*. This *CogFit* checklist was newly developed by our research team.

## Methods

### Development of the CogFit checklist

#### Identification of risk/protective factors

We initially extracted relevant factors from the report by the Lancet Commission on Dementia Prevention in 2020^3^ and the report from the World Health Organization (WHO)^2^; both providing a consensus on the current state of evidence. As this scientific field is very active, we conducted an additional search for new literature in PubMed and Google Scholar on January 13th, 2023 using the search string (risk factors OR modifiable factors OR lifestyle OR risk score) AND (dementia OR Alzheim* OR “neurocognitive disorder” OR “neurocognitive disorders”). We obtained *n* = 220 search hits in PubMed and *n* = 466 in Google Scholar. Only publications that were (i) meta-analyses and systematic reviews, (ii) published between 2020 and 2023, (iii) in English, (iv) with an abstract, and (v) providing evidence on the association of risk and protective factors on dementia incidence were considered appropriate. After screening, *n* = 42 publications turned out to be relevant. Excluded publications regarded mostly COVID-19, neurological characteristics of dementia patients, genetics, or interventions for people with dementia. From these 42 publications as well as the Lancet^3^ and WHO^4^ sources, we extracted the following information in an Excel spreadsheet: factor(s) examined, significance of the results, and effect size (HR, OR, RR). Further, based on the authors’ knowledge, we searched for sleep disturbances by hand. A meta-analysis confirmed evidence on the role of sleep disturbances on brain health^15^, and their modifiability led us to include it.

#### Selection of risk/protective factors

Using the list of risk factors identified in the previous step, two scientists in the field of dementia independently and blinded to the ratings of each other ranked the factors by (i) effect size and (ii) modifiability. Regarding effect sizes, the factor for which studies reported the largest effect sizes was assigned the highest rank and the factor for which studies reported the smallest effect sizes was assigned the lowest rank. Regarding modifiability, the factor that is most easily modified for a person (as considered by the rater) was assigned the highest rank and the factor that is least possibly to be modified by an individual was assigned the lowest rank. As the ranges varied between raters, percentiles were calculated. The correlation between the raters was high (effect size: Spearman’s rho = 0.872, *p* < 0.001, average ICC = 0.929, CI95% 0.878–0.959, *p* < 0.001; modifiability: Spearman’s rho = 0.767, *p* < 0.001, average ICC = 0.860, CI95% 0.767–0.916, *p* < 0.001). We averaged the ratings of the two raters and sorted the factors by rank. As the checklist was meant to be only one page (for reasons of practicality), we excluded all those scoring below the top 50 for either effect size or modifiability. We further excluded *education*,* environmental atmosphere*,* cerebral microbleeds*,* coronary artery bypass grafting*, and f*railty* because these factors cannot be modified on an individual level. We also excluded *benzodiazepines use*,* vitamin B status*, and *anticholinergic medication* as these factors might not be immediately known by ageing individuals. See Supplementary File 1 for details.

#### Checklist formatting

We formulated questions for each factor. The wording was chosen so that it indicates the presence of the factors, patients over 60 years old can answer independently, and the answers can easily be interpreted by the GPs. A first version of the *CogFit* checklist was reviewed internally by researchers. Feedback regarding wording and coloring to associate protective or detrimental effects was incorporated.

### Sample

The Ethics Committee of the University Medical Center Greifswald, Germany (BB 065/23) approved the study. The study was conducted in accordance with the Declaration of Helsinki. All participants provided written informed consent before enrollment.

The only inclusion criteria were being an actively practicing GP and having signed the informed consent. GPs were recruited in a 100 km radius around our institute in the state of Mecklenburg–Western Pomerania, Germany, from June to September 2023. We visited a total of *n* = 107 GPs that were identified online (GP website or healthcare platform). Eighteen GPs agreed to participate. GPs received copies of the *CogFit* checklist as well as the GP experience questionnaire. They were instructed to test the checklist with at least three of their patients who did not have dementia and fill out questionnaires on their experience after approximately one month. Completed GP experience questionnaires were returned to us and the GPs received a compensation of 200 Euros. The completed *CogFit* checklists remained with the patients; their output was were not part of this initial study. We collected answers up to January 2024.

### GP experience questionnaires

We provided GPs with two questionnaires, one developed specifically for this study and the System Usability Scale (SUS)^16^. To create the study-specific GP experience questionnaire, we screened publications on similar studies and included items relevant to using the checklist. The final questionnaire included personal information (gender, age, years working), details on the use of the *CogFit* checklist (number of patients who received it, compliance, completion modalities, problems, information obtained, actions taken), embedding in GP office processes (impact on treatment, prior knowledge, lack of treatment options), need for improvement (format of the checklist and its evaluation, preferences, additional needs), and usability (perceived usefulness, willingness to use in future). For each aspect, questions were formulated. The questionnaire contained 16 multiple choice questions, ten number questions, seven 5-point Likert-scale (strongly disagree (1) - strongly agree (5)) questions, and ten open-ended questions (see Supplementary File 2).

In addition, we used the SUS, developed by John Brooke (1996) for contextual analyses of usability^16^. It contains ten items on a five-point Likert scale (strong disagreement (1) to strong agreement (5)). To achieve a consistent scale of 0–4 for all questions, we subtracted one for positive questions and subtracted the score from five for negative ones. The sum score was multiplied by 2.5 to get our final score (range 0–100), with higher scores representing better usability.

### Analyses

We calculated the frequencies and/ or means of the answers of multiple-choice, number, and Likert-scale questions. For open-ended questions, we reported the GPs’ answers as text. We estimated the associations between willingness to use the *CogFit* checklist in the future with several variables, i.e., the number of patients the checklist was tested with, GP age, perceived usefulness of the checklist, and perceived time-effort via Spearman ranks correlation bootstrapped with 1000 replications.

## Results

The patient self-completion checklist *CogFit* contained 24 modifiable risk and protective factors that we extracted from our literature search (Supplementary File 1). Factors absent in the Lancet and WHO lists were psychological disorders, kidney disease, chronic pain, periodontitis, stress, having a purpose in life, playing a musical instrument, and coffee/green tea consumption (see Table 1).

**Table 1:** Risk/protective factors in *CogFit*, and other lists of dementia risk factors. *CogFit* was built based on literature reviewed in January 2023 with the aim of detecting modifiable risk factors in primary care that can be acted on.

Factor	CogFit	Lancet commission (Livingston et al. 2024)	World health organization (2019)	LIBRA score (Rosenau et al. 2024)	CogDrisk (Anstey et al. 2022)
Physical activity	x	x	x	x	x
Obesity	x	x	x	x	x
Hypertension	x	x	x	x	x
Social isolation	x	x	x	x	x
Depression	x	x	x	x	x
Smoking	x	x	x	x	x
Diabetes	x	x	x	x	x
Alcohol abuse	x	x	x	x	
Hearing impariment	x	x	x	x	
Mediterreanan diet	x		x	x	x
cognitive activities	x		x	x	x
Sleep problems	x			x	x
Renal dysfunction	x			x	
Dental problems	x				
Chronic pain	x				
Stress	x				
Coffee/ green tea consumption	x				
Purpose in life	x				
Not included in *CogFit* as not ranked among high enough (modifiability/ effect size)					
Traumatic brain injury		x			x
Air pollution		x			
Not included in *CogFit* as not modifiable in old age					
Education		x			x
Heart disease				x	x
Not included in *CogFit* as not among the literature search hits					
Vision loss		x			
High cholesterol		x	x	x	x
Stroke					x
Pesticide exposure					x

GPs were requested to use the checklist for identifying risk factors for dementia among their patients. A total of 14 GPs had filled out the questionnaires on their experiences (response rate 13.1%). They were, on average, 44.8 (SD = 7.8) years old and 71.4% female; 21.4% had less than ten years of work experience, 57.1% had between 10 and 20 years of work experience, and 21.4% had more than 20 years of work experience. Details on their responses are shown in Supplementary File 2.

GPs distributed the *CogFit* checklist on average to 5.9 (SD = 3.3, total *n* = 83) patients. 69.2% of the GPs reported that all patients they asked to complete the checklist did so. Two GPs said that about 2% and another two GPs that about 20% of their patients refused to complete it. Half of the GPs had their patients complete the *CogFit* checklist in the waiting room and 14.3% at home. All GPs indicated sufficient time to complete the checklist in their practice, and almost two-thirds of them (64.3%) reported to have discussed the checklist with their patients in a personal conversation.

### Details of use

GPs reported that their patients answered, on average, 98.0% (SD = 4.2%) of the questions. Omissions regarded alcohol consumption, stress, health state, and playing a musical instrument. While one third of the GPs (28.6%) did not witness any problems, others reported patients misunderstanding questions (35.7%), patients’ difficulties in reading (21.4%), or needing time to think (14.3%). In free-text questions, GPs described the *CogFit* checklist as unsuitable for people with severe dementia, older than 90 years, living in nursing homes, with severe depression, severe intellectual disability, and whose mother tongue was not German.

Most GPs (71.4%) gained important information about their patients, specifically regarding social contacts (28.6%), depression, sleep disorders, leisure and mental activities (14.3% for each), purpose in life, hearing, physical exercise, and nutrition (by one GP each). Consequent actions taken by the GPs included lifestyle advice (69.2%) for most patients (average across GPs 65.0%) and referral to brochures or courses (61.5%) for many patients (average across GPs 55.8%). Less frequent was a referral to a specialist/ therapist, medication prescription, referral to rehabilitation/ prevention programs, or health insurance programs (Table 2).

**Table 2:** Actions taken by GPs based on the checklist outcome.

Action taken	GPs taking action	GPs’ answer to the question “For what percentage of the patients did you take this action?”
	% (n)	mean (SD) over all participating GPs
Lifestyle advice	69.2 (9)	65.0% (29.3)
Referral to brochures or courses	61.5 (8)	55.8% (38.4%)
Referral to specialist/ therapist	38.5 (5)	62.5% (45.0%)
Prescribed medications	30.8 (4)	3.3% (5.8%)
Rehabilitation/ prevention course	15.4 (2)	26.5% (9.2%)
Programs by health insurances	7.1 (1)	no information

**Fig. 1:**
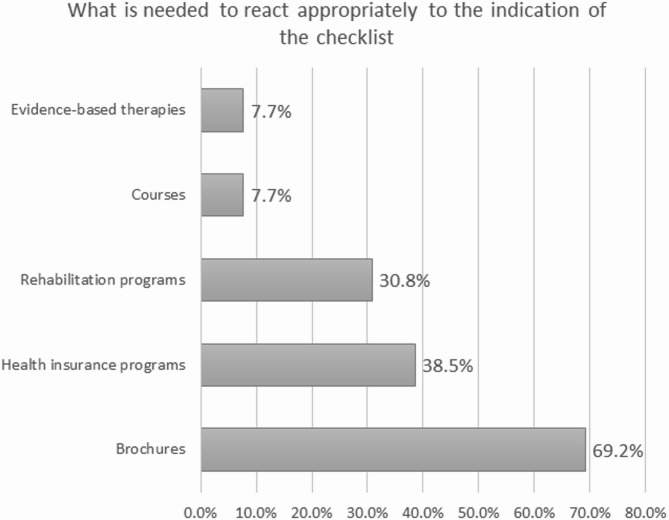
Responses by the GPs to the question on what is needed to react appropriately to the indication of the checklist.

Participating GPs mentioned that brochures (69.2%), health insurance programs (38.5%), rehabilitation/ prevention programs (30.8%), and courses (one GP) are missing but yet needed to take appropriate actions (see also Fig. 1). In free-texts, GPs expressed specific wishes for (i) brochures and courses informing about healthy nutrition and dementia, (ii) health insurance programs to deal with dementia and with activities to stay fit in old age, (iii) rehabilitation/prevention programs specifically for dementia, (iv) ‘evidence-based therapies’ (not further explained), and (v) courses to learn/play musical instruments.

### Embedding in GP office processes

Concerning the checklist-associated workload, 28.6% of the GPs reported no additional, 35.7% a moderately increased, and 21.4% an extremely increased workload (for the specific wording used by the GPs, see Supplementary file 2). In free-texts, GPs reported that reasons for the increased workload included explaining to patients why they should complete it, explaining the questions, evaluating the checklist, and discussing the patients’ answers. Only 42.9% of the GPs reported using other checklists in their practice, mainly for health status assessments (e.g., Barthel Index, Mini Mental Status Test, Geriatric Depression Scale, Wells Score).

**Fig. 2:**
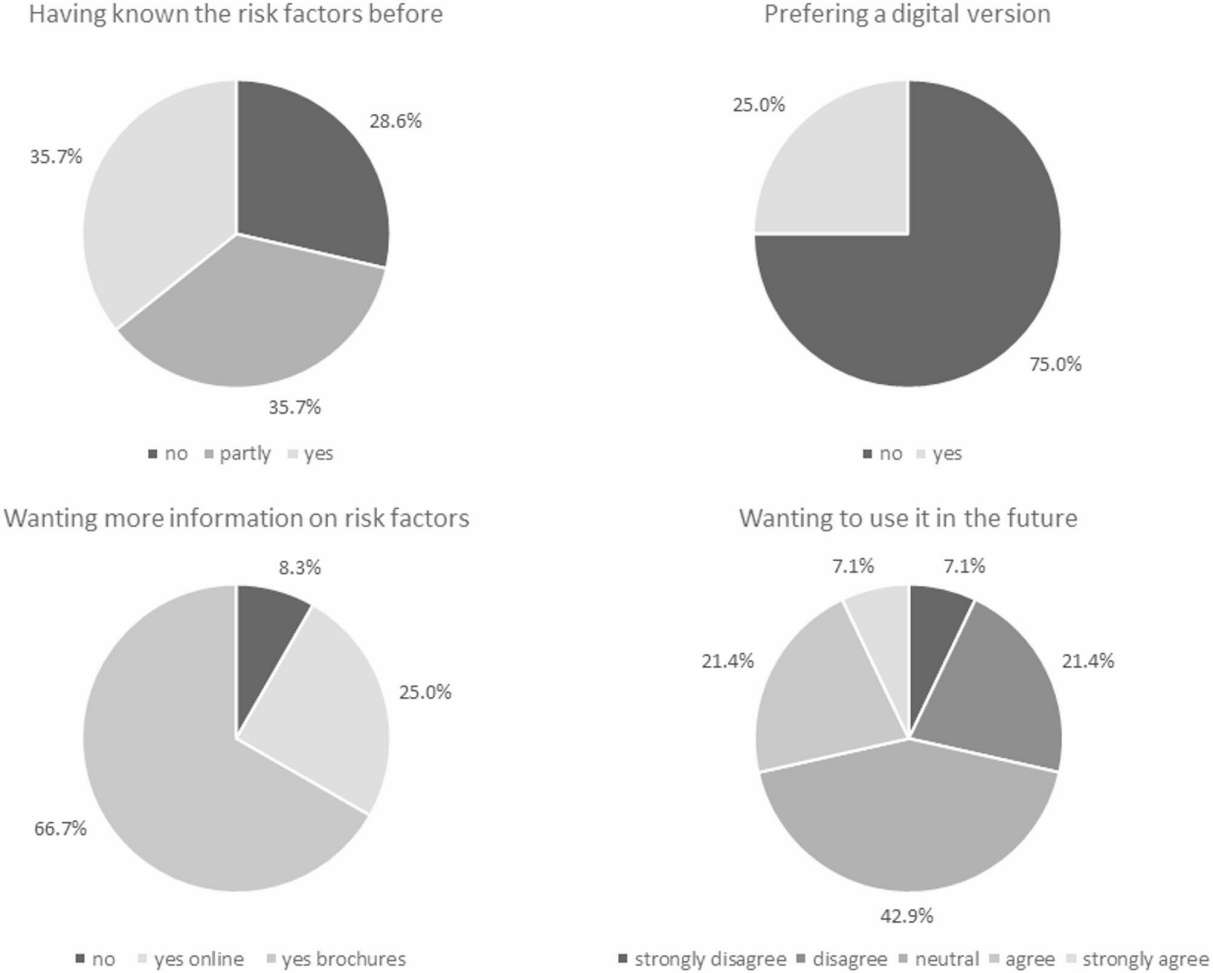
Percentages of GPs reporting whether they have known the risk factors, want more information on risk factors, prefer a digital version, and whether they want to use it in the future (lighter areas indicate agreement; darker areas indicate disagreement).

Despite most GPs (71.4%) being aware of the dementia risk and protective factors on the *CogFit* checklist, they wished to have additional information on how to handle them in the form of brochures (66.7%) or online events (25.5%; see also Fig. 2). Concerning chronic diseases on the checklist, 64% of the GPs indicated that they have no methods of monitoring these diseases regularly.

### Feedback for improvement

When GPs were asked whether risk factors were missing, three GPs mentioned ‘personal hygiene’, ‘medication’, and ‘personal wishes’ (without further explanation). Only three GPs said a digitized version of the *CogFit* checklist would be useful.

Regarding the evaluation of the checklist, ten GPs (83%) considered the current version sufficient. As the *CogFit* checklist only included modifiable factors, we asked the GPs if they think that non-modifiable risk factors should be included. They tended to disagree (M = 2.5, SD = 1.1, on a Likert-scale from completely disagree (1) to completely agree (5)). Rather, they favored simplifying the language (M = 3.6, SD 1.3) and using visual aids (M = 3.5, SD 1.3). Overall, GPs suggested (i) adding a graphic representation of the results, (ii) adding a score with a cutoff that signals if an in-depth conversation should take place, (iii) specifying that physicians’ assistants should complete the checklist with the patient and then consult the GP on consequent recommendations, and (iv) integrating the checklist into regular health checks. Concerning the wording, negative expressions (e.g., lack of something or “less than”) were sometimes misunderstood. One GP complained about too long or too general questions.

Based on this feedback, we improved the *CogFit* checklist by inverting the direction of negative questions (e.g., “less than two glasses” to “two or more glasses”; Q3, Q4, Q5, Q6, Q7, Q8) and shortening long ones (e.g., “Have you been diagnosed with hearing impairment or do other people say that your hearing has gotten worse?” to “Do you have a hearing impairment? (e.g., diagnosis or others say you hear badly)”; Q11, Q13). Since we targeted only evidence-based factors, we did not change the content of the checklist (see Supplementary file 3).

### Usability

57% of GPs explicitly considered the *CogFit* checklist useful and 21.4% were neutral. They ‘slightly disagreed’ that it takes too much time (M = 2.6, SD = 1.4). Regarding their intent to use the *CogFit* checklist in the future, four GPs agreed (28.6%), four disagreed, and six (42.9%) remained neutral (see also Fig. 2). There was no statistically significant correlation between willingness to use the checklist in the future and the number of patients the checklist was tested with (rho = 0.106, 95% confidence interval (CI95%) -0.499;0.711, *p* = 0.732) or the perceived time effort (rho=-0.425, CI95% -0.914;0.063, *p* = 0.088). GPs who perceived the checklist as useful were more willing to use it in the future (rho = 0.761, CI95% 0.489;1.03, *p* < 0.001). Older GPs were significantly less favorable than younger GPs (rho=-0.563, CI95% -0.991;-136, *p* = 0.010).

**Fig. 3:**
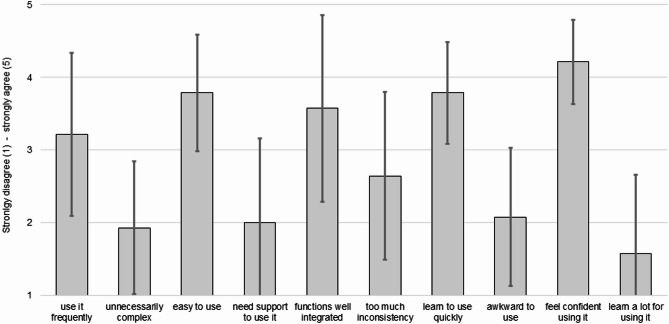
Means and standard deviations of the GPs’ answers to the System Usability Scale (SUS) questions on the usability of the checklist.

With an average SUS score of 73.13 (SD = 10.45), the user-friendliness of the *CogFit* checklist was good. The questions on *feeling confident to use* (M = 4.21, SD = 0.58), *ease of use (*M = 3.79, SD = 0.80), and *learning quickly to use the checklist* (M = 3.79, SD = 0.69) met the highest agreement, while the lowest agreement was found for the items *having to learn a lot for using it* (M = 1.57, SD = 1.09), feeling that it is *unnecessarily complex* (M = 1.93, SD = 0.92), and that *support is needed to use the checklist* (M = 2.00, SD = 1.15, see Fig. 3). Perceived usability (SUS score) was not associated with willingness to use the checklist in the future (rho = 0.077, CI95% -0.669;0.823, *p* = 0.839).

## Discussion

In this study, we examined a new approach to dementia prevention involving GPs. Specifically, we investigated the practicality of using a patient self-completion screening checklist of modifiable dementia risk and protective factors (*CogFit)*, which was developed by our research team. GPs considered the checklist useful and easy to use. They indicated that it provided information leading to action. This supports the practicality of the approach. However, more steps are necessary to establish effectiveness. GPs reported gaps in materials and services (e.g., brochures, courses) that would make this checklist more actionable. Even though brochures informing about risk factors (e.g.,^17^ are already available, material that supports lifestyle changes with practical advice is still rare. These, however, are needed for future research with *CogFit*. Enhancing the availability of such materials as well as algorithms to act on the indications of the checklist may increase GPs’ willingness to use it in the future. Moreover, if GPs had tutorials on risk factor improvement for their patients or if long-term collaborations with existing programs/ community stakeholders were established, it would be easier for GPs to help their patients. Furthermore, a digital version of *CogFit* (i.e., smartphone app) could empower patients themselves and in this way unburden GPs.

Checklists to screen for dementia are used by GPs^18^, but they seem to be missing for detecting risk factors. Dementia risk scores used in academic clinics include clinical markers (e.g., APOE e4 gene, biomarkers, imaging) that are often not available to GPs and do not focus on lifestyle^19^. New mobile health applications to assess modifiable risk factors are starting to be developed and promise high usability^20^, for example the ‘*Platform for Dementia Prevention in the Prevention of Dementia by Mobile Phone Applications’* (PRODEMOS). Despite needed improvement^21^, interest for using mobile dementia risk reduction tools^22^ is a good starting point. However, using an app depends on individuals’ voluntary initiative. Evidence on voluntary health checkups emphasizes that people with higher socioeconomic status and healthier lifestyle participate more regularly in health checkups^23^ For dementia prevention, it is crucial to reach more those who have the highest risk, which tend to be those who do not seek prevention. GPs are the first contact for most people in the society and their advice is generally trusted^24^.

Risk factors screening involving GPs is rare. In Australia, a *GP Dementia Risk Reduction Education* project was started to enhance hypertension screening and offer education workshops^25^ This is an important first step, but not comprehensive. A review of primary care guidelines concluded that dementia risk reduction should involve discussions with patients, identification of patients at risk, managing dementia risk factors, offering additional support, and regular follow-ups^26^ Integrating a tool like *CogFit* in regular GP care would enable precisely those steps. Our GPs provided positive feedback in many respects. Nonetheless, about a third of the GPs were not willing to use the *CogFit* checklist in the future. A lower willingness was associated with older age. It may also relate to a lack of dedicated compensation. Methods of reimbursement will have to be discussed with health insurances for future research. Compliance might also be better if risk factor screening was a regular part of nationwide health insurance programs. Providing GPs with relevant information on risk factors, as they wish, might help them understand the necessity for it. Evidence from research on other chronic conditions (e.g., cardiovascular risk factors, cancer, diabetes) indicates that, even though many GPs see prevention as one of their responsibilities,^27^ GPs’ willingness to screen for risk factors is higher if GPs think that the patient’s absolute risk is high^28^, that patients will make lifestyle changes^29^, and whether time is available for the assessment^28^. Yet, empowering patients by providing them personally with the checklist could indirectly enhance a GPs’ motivation to engage in risk factor screening as patients may want to discuss their situation with them. New prevention initiatives can be difficult to implement. The English “NHS Health Check” to reduce cardiovascular disease (by prescribing medication, referring to smoking cessation programs and, in some cases, offering lifestyle support) reached only 21.4% of the population^30^ Still, participating patients improved significantly in blood pressure, cholesterol, and blood lipid levels,^31^ with an estimated overall cardiovascular risk reduction from 32.9–29.4%.^32^ Analogous improvements could be expected for cognitive health.

Our *CogFit* checklist intends to tailor preventive actions by immediately prompting tangible behaviors that benefit the general population’s health and reduce dementia risk on a large scale. While multi-domain intervention programs such as the FINGER study^6^ and the German AgeWell study^33^ encompass a broad range of actions, they are generally too expensive to be implemented in the general population. Instead of targeting all potential risk factors, focusing on the most problematic risk factor(s) of a person might be a more practical approach. The Alzheimer’s Prevention Clinic (APC) in New York started an initiative entailing intensive medical screening and tailored interventions (i.e., patient education, genetic counselling, pharmacological approaches, recommendations for exercise, nutrition, sleep, cognitive engagement, stress, etc.). They observed improvements in cognitive functioning^34^ While their findings are promising, the setting and range of actions is different than those of GP practices. Future research will have to determine to what extent the tailored approach with *CogFit* can lead to longitudinal changes in risk factor prevalence and cognitive functioning.

The study comes with strengths and limitations. One limitation is certainly that, in this initial step, we recruited only a small number of GPs. This was sufficient for the goal of assessing the practicality of the checklist. However, some of these GPs tested the *CogFit* checklist with only few patients. In this case, their reports might be biased due to the characteristics of those specific patients. Second, the *CogFit* checklist was tested in a rural state in Germany. It is conceivable that the results might differ in urban areas and in other countries. Third, we collected data from GPs only, and not from patients. In that respect, patient outcomes (e.g., depression, cognitive performance) were not assessed and algorithms leading from the indication to change in patient outcome or behavior could not be estimated. Moreover, benefits associated with using the checklist may differ between cognitively intact people, those with mild cognitive impairment, and those in early stages of dementia, but all may benefit from such screening and the consequent preventive action, and may therefore be also targeted in next studies. A future study should measure patient-specific behavioral modifications and longitudinal changes in cognitive functioning, health status, and well-being in these groups. Within the context of such a study, these should be assessed using standardized instruments (e.g., neurological test battery) and questionnaires (e.g., symptom checklists, quality of life indicators, psychological self-report forms). Of course, considerations regarding reliability and power must be taken in account when planning such a study. For example, a sample size that compares the effectiveness of using the checklist with usual care needs to have more than *n* = 1,000 participants if the observed difference in means is 0.2 or less. Moreover, for generalizability, it is important that such a study is conducted where the majority of the population receives primary care (not in specialized clinics). Indications that can be derived from such a study (e.g., what aspects of the checklist have the strongest impact on patients, patient feedback) will guide the further development of the checklist. Fourth, some modifiable dementia risk and protective factors may not be included in the *CogFit* checklist. Despite a literature review, we might have omitted factors that were not captured by our search terms or that are still under investigation. A periodical update of the checklist is recommended. Expanding the use of the checklist to other settings (e.g., clinics) may allow for the inclusion of further risk factors.

## Conclusions

This work shows the practicality of a prevention pathway that involves GPs. While the effectiveness of using the *CogFit* checklist in primary care still has to be demonstrated with patients, a definite advantage of the approach is that the completion of the checklist by patients themselves empowers them to take an active role in the process. To our knowledge, our checklist is the first tool for detecting modifiable dementia risk and protective factors specifically created for and tested in GP practices. Overall, such a prevention approach could be an optimal strategy for dementia prevention, probably better than focusing on high-risk patients alone^35^.

## Electronic supplementary material

Below is the link to the electronic supplementary material.

Supplementary Material 1

Supplementary Material 2

Supplementary Material 3

## Data Availability

The datasets generated and analyzed during the current study are not publicly available due restrictions in the informed consent. For eligible data requests contact the corresponding author (F. Rodriguez, Email francisca-saveria.rodriguez@dzne.de).

## References

[1] Grande, G., Qiu, C. & Fratiglioni, L. Prevention of dementia in an ageing world: evidence and biological rationale. *Ageing Res. Rev.* **64**, 101045 (2020). 32171784 10.1016/j.arr.2020.101045

[2] Organization, W. H. *Risk Reduction of Cognitive Decline and Dementia: WHO Guidelines* (World Health Organization, 2019). 31219687

[3] Livingston, G. et al. Dementia prevention, intervention, and care: 2020 report of the lancet commission. *Lancet* **396**, 413–446 (2020). 32738937 10.1016/S0140-6736(20)30367-6 PMC7392084

[4] Organization, W. H. *Global Action Plan on the Public Health Response To Dementia 2017–2025* (World Health Organization, 2017).

[5] Marengoni, A. et al. The effect of a 2-Year intervention consisting of diet, physical exercise, cognitive training, and monitoring of vascular risk on chronic Morbidity-the FINGER randomized controlled trial. *J. Am. Med. Dir. Assoc.* **19**, 355–360 (2018). e351. 29108888 10.1016/j.jamda.2017.09.020

[6] Ngandu, T. et al. A 2 year multidomain intervention of diet, exercise, cognitive training, and vascular risk monitoring versus control to prevent cognitive decline in at-risk elderly people (FINGER): a randomised controlled trial. *Lancet* **385**, 2255–2263 (2015). 25771249 10.1016/S0140-6736(15)60461-5

[7] Kivipelto, M. et al. A global approach to risk reduction and prevention of dementia. *Alzheimers Dement.* **16**, 1078–1094 (2020). World-Wide FINGERS Network. 32627328 10.1002/alz.12123 PMC9527644

[8] Hafdi, M., Hoevenaar-Blom, M. P. & Richard, E. Multi-domain interventions for the prevention of dementia and cognitive decline. *Cochrane Database Syst. Rev.* **11**, CD013572 (2021). 34748207 10.1002/14651858.CD013572.pub2 PMC8574768

[9] Walsh, S. et al. A systematic review of the cost-effectiveness of community and population interventions to reduce the modifiable risk factors for dementia. *Maturitas* **166**, 104–116 (2022). 36150253 10.1016/j.maturitas.2022.09.002

[10] Mukadam, N. et al. Effective interventions for potentially modifiable risk factors for late-onset dementia: a costs and cost-effectiveness modelling study. *Lancet Healthy Longev.* **1**, e13–e20 (2020). 36094185 10.1016/S2666-7568(20)30004-0

[11] Anstey, K. J., Kootar, S., Huque, M. H., Eramudugolla, R. & Peters, R. Development of the CogDrisk tool to assess risk factors for dementia. *Alzheimers Dement. (Amst)*. **14**, e12336 (2022). 35845259 10.1002/dad2.12336 PMC9275658

[12] Dolphin, H. et al. New horizons in the diagnosis and management of Alzheimer’s disease in older adults. *Age Ageing*. **53**, afae005 (2024). 38342754 10.1093/ageing/afae005 PMC10859247

[13] Rockwood, K., McMillan, M., Mitnitski, A. & Howlett, S. E. A frailty index based on common laboratory tests in comparison with a clinical frailty index for older adults in Long-Term care facilities. *J. Am. Med. Dir. Assoc.* **16**, 842–847 (2015). 25952475 10.1016/j.jamda.2015.03.027

[14] Geffen, L. N., Kelly, G., Morris, J. N. & Howard, E. P. Peer-to-peer support model to improve quality of life among highly vulnerable, low-income older adults in cape town, South Africa. *BMC Geriatr.* **19**, 1–12 (2019). 31640576 10.1186/s12877-019-1310-0 PMC6805367

[15] Shi, L. et al. Sleep disturbances increase the risk of dementia: A systematic review and meta-analysis. *Sleep. Med. Rev.* **40**, 4–16 (2018). 28890168 10.1016/j.smrv.2017.06.010

[16] Brooke, J. SUS-A quick and dirty usability scale. *Usability Evaluation Ind.* **189**, 4–7 (1996).

[17] Initiative, A. F. *Alzheimer-Demenz Vorbeugen* (Alzheimer Forschung Initiative, Düsseldorf, 2022).

[18] Milne, A., Culverwell, A., Guss, R., Tuppen, J. & Whelton, R. Screening for dementia in primary care: a review of the use, efficacy and quality of measures. *Int. Psychogeriatr.* **20**, 911–926 (2008). 18533066 10.1017/S1041610208007394

[19] Sindi, S. et al. The CAIDE dementia risk score app: the development of an evidence-based mobile application to predict the risk of dementia. *Alzheimers Dement. (Amst)*. **1**, 328–333 (2015). 27239514 10.1016/j.dadm.2015.06.005 PMC4878198

[20] Reid, G. et al. The usability and reliability of a smartphone application for monitoring future dementia risk in ageing UK adults. *Br. J. Psychiatry*. **224**, 245–251 (2024). 38356396 10.1192/bjp.2024.18 PMC11443166

[21] Hafdi, M. et al. Design and development of a mobile health (mHealth) platform for dementia prevention in the prevention of dementia by mobile phone applications (PRODEMOS) project. *Front. Neurol.* **12**, 733878 (2021). 34975710 10.3389/fneur.2021.733878 PMC8716458

[22] O’Connor, E., Farrow, M. & Hatherly, C. Randomized comparison of mobile and Web-Tools to provide dementia risk reduction education: use, engagement and participant satisfaction. *JMIR Ment Health*. **1**, e4 (2014). 26543904 10.2196/mental.3654 PMC4607394

[23] Hoebel, J., Starker, A., Jordan, S., Richter, M. & Lampert, T. Determinants of health check attendance in adults: findings from the cross-sectional German health update (GEDA) study. *BMC Public. Health*. **14**, 913 (2014). 25185681 10.1186/1471-2458-14-913 PMC4167266

[24] SLAMA, K. J., REDMAN, S., COCKBURN, J. & SANSON-FISHER, R. W. Community views about the role of general practitioners in disease prevention. *Fam. Pract.* **6**, 203–209 (1989). 2792621 10.1093/fampra/6.3.203

[25] Phillipson, L. & Jones, S. C. The GP Dementia Risk Reduction Education Project: Literature Review and Formative Research with General Practitioners. (2012).

[26] Godbee, K. et al. Dementia risk reduction in primary care: A scoping review of clinical guidelines using a behavioral specificity framework. *J. Alzheimers Dis.* **89**, 789–802 (2022). 35938252 10.3233/JAD-220382 PMC9697048

[27] Holmberg, C. et al. Primary prevention in general practice – views of German general practitioners: a mixed-methods study. *BMC Fam. Pract.* **15**, 103 (2014). 24885100 10.1186/1471-2296-15-103 PMC4046439

[28] Bonner, C. et al. General practitioners’ use of different cardiovascular risk assessment strategies: a qualitative study. *Med. J. Aust.* **199**, 485–489 (2013). 24099210 10.5694/mja13.10133

[29] Burridge, L., Mitchell, G., Jiwa, M. & Girgis, A. Helping Lay carers of people with advanced cancer and their gps to talk: an exploration of Australian users’ views of a simple carer health checklist. *Health Soc. Care Commun.* **25**, 357–365 (2017). 10.1111/hsc.12312 26694537

[30] Chang, K. C. M. et al. Coverage of a National cardiovascular risk assessment and management programme (NHS health Check): Retrospective database study. *Prev. Med.* **78**, 1–8 (2015). 26051202 10.1016/j.ypmed.2015.05.022

[31] Artac, M., Dalton, A. R. H., Majeed, A., Car, J. & Millett, C. Effectiveness of a National cardiovascular disease risk assessment program (NHS health Check): Results after one year. *Prev. Med.* **57**, 129–134 (2013). 23701848 10.1016/j.ypmed.2013.05.002

[32] Cochrane, T. et al. NHS health checks through general practice: randomised trial of population cardiovascular risk reduction. *BMC Public. Health*. **12**, 944 (2012). 23116213 10.1186/1471-2458-12-944 PMC3524756

[33] Zülke, A. et al. AgeWell.de - study protocol of a pragmatic multi-center cluster-randomized controlled prevention trial against cognitive decline in older primary care patients. *BMC Geriatr.* **19**, 203 (2019). 31370792 10.1186/s12877-019-1212-1 PMC6670136

[34] Isaacson, R. S. et al. Individualized clinical management of patients at risk for Alzheimer’s dementia. *Alzheimers Dement.* **15**, 1588–1602 (2019). 31677936 10.1016/j.jalz.2019.08.198 PMC6925647

[35] Zulman, D. M., Vijan, S., Omenn, G. S. & Hayward, R. A. The relative merits of population-based and targeted prevention strategies. *Milbank Q.* **86**, 557–580 (2008). 19120980 10.1111/j.1468-0009.2008.00534.x PMC2690369

