# Supplementary material for: Practicality of a patient self-assessment checklist to manage dementia risk factors in GP practices
Source: Sci Rep. 2025 May 16;15:17064. doi: 10.1038/s41598-025-01455-8 (PMC12084373; doi:10.1038/s41598-025-01455-8)
Supplement: Supplementary file 1 — Supplementary Material 1 [file 41598_2025_1455_MOESM1_ESM.pdf]

Supplementary File 1

To the manuscript ‘Practicality of a patient self-assessment checklist to manage dementia risk factors in GP practices’ (Rodriguez et al.)

Identified risk and protective factors that were included and not included in the checklist.

#	Risk/ protective factor	Question in checklist	Reference	Effect (extracted from the publication)	Average rating: %-rank effect size	Average rating: %-rank modify-ablity
**Included in checklist**						
1	Alcohol abuse	Q8	Litke R, Garcharna LC, Jiwani S, Neugroschl J. Modifiable risk factors in Alzheimer disease and related dementias: a review. Clinical Therapeutics. 2021;43(6):953-965.	RR=0.74, CI 95% 0.61, 0.91	48	22
			Livingston, G., Huntley, J., Sommerlad, A., Ames, D., Ballard, C., Banerjee, S., ... & Mukadam, N. (2020). Dementia prevention, intervention, and care: 2020 report of the Lancet Commission. The Lancet, 396(10248), 413-446.	RR=1.2, CI 95% 1.1, 1.3	45	37
			World Health Organization. (2019). Risk reduction of cognitive decline and dementia: WHO guidelines.	“Interventions aimed at reducing or ceasing hazardous and harmful drinking should be offered”	45	37
2	Anxiety	Q17	Kuring JK, Mathias JL, Ward L. Risk of Dementia in persons who have previously experienced clinically-significant Depression, Anxiety, or PTSD: A Systematic Review and Meta-Analysis. J Affect Disord. 2020;274:247-261.	OR=1.60, CI 95% 1.29, 2.00	37	47
3	Chronic pain	Q14	Yuan H, Ahmed WL, Liu M, Tu S, Zhou F, Wang S. Contribution of pain to subsequent cognitive decline or dementia: A systematic review and meta-analysis of cohort studies. Int J Nurs Stud. 2022;138:104409.	OR=1.29, CI 95% 1.17, 1.41	65	29
4	Coffee	Q4	Mentis AA, Dardiotis E, Efthymiou V, Chrousos GP. Non-genetic risk and protective factors and biomarkers for neurological disorders: a meta-umbrella systematic review of umbrella reviews. BMC Med. 2021;19(1):6.	RR=0.73, CI 95% 0.54, 0.99; RR=0.73, CI 95% 0.55, 0.97	-	1
5	Cognitive stimulation workplace	Q1	Kivimäki M, Walker KA, Pentti J, et al. Cognitive stimulation in the workplace, plasma proteins, and risk of dementia: three analyses of population cohort studies. Bmj. 2021;374:n1804.	HR=0.60, CI 95% 0.37, 0.95	31	65
	Cognitive activity	Q1	Yu JT, Xu W, Tan CC, et al. Evidence-based prevention of Alzheimer's disease: systematic review and meta-analysis of 243 observational prospective studies and 153 randomised controlled trials. J Neurol Neurosurg Psychiatry. 2020;91(11):1201-1209.	RR=0.050, CI 95% 0.39, 0.63	29	6
	Cognitive training	Q1	World Health Organization. (2019). Risk reduction of cognitive decline and dementia: WHO guidelines.	“Cognitive training may be offered”	13	10

#	Risk/ protective factor	Question in checklist	Reference	Effect (extracted from the publication)	Average rating: %-rank effect size	Average rating: %-rank modify-ablity
6	Delusional disorder	Q17	Miniawi SE, Orgeta V, Stafford J. Non-affective psychotic disorders and risk of dementia: a systematic review and meta-analysis. Psychol Med. 2022;52(15):1-13.	RR=2.52, CI 95% 1.67, 3.89	3	85
7	Depression	Q17	Dragioti E, Radua J, Solmi M, et al. Global population attributable fraction of potentially modifiable risk factors for mental disorders: a meta-umbrella systematic review. Mol Psychiatry. 2022;27(8):3510-3519.	RR=1.83, CI 95% 1.65, 2.03	24	36
			Kuring JK, Mathias JL, Ward L. Risk of Dementia in persons who have previously experienced clinically-significant Depression, Anxiety, or PTSD: A Systematic Review and Meta-Analysis. J Affect Disord. 2020;274:247-261.	OR=2.23, CI 95% 1.46, 3.41	17	36
			Yu JT, Xu W, Tan CC, et al. Evidence-based prevention of Alzheimer's disease: systematic review and meta-analysis of 243 observational prospective studies and 153 randomised controlled trials. J Neurol Neurosurg Psychiatry. 2020;91(11):1201-1209.	RR=1.80, CI 95% 1.34, 2.42	25	36
			Litke R, Garcharna LC, Jiwani S, Neugroschl J. Modifiable risk factors in Alzheimer disease and related dementias: a review. Clinical Therapeutics. 2021;43(6):953-965.	HR=1.94, CI 95% 1.30, 2.90	14	36
			Stafford J, Chung WT, Sommerlad A, Kirkbride JB, Howard R. Psychiatric disorders and risk of subsequent dementia: Systematic review and meta-analysis of longitudinal studies. Int J Geriatr Psychiatry. 2022;37(5).	RR=1.96, CI 95% 1.59, 2.43	12	36
			Livingston, G., Huntley, J., Sommerlad, A., Ames, D., Ballard, C., Banerjee, S., ... & Mukadam, N. (2020). Dementia prevention, intervention, and care: 2020 report of the Lancet Commission. The Lancet, 396(10248), 413-446.	RR=1.9, CI 95% 1.6, 2.3	21	37
			Mentis AA, Dardiotis E, Efthymiou V, Chrousos GP. Non-genetic risk and protective factors and biomarkers for neurological disorders: a meta-umbrella systematic review of umbrella reviews. BMC Med. 2021;19(1):6.	RR=1.99, CI 95% 1.84, 2.16; RR=1.77 1.48, 2.13	21	37
			World Health Organization. (2019). Risk reduction of cognitive decline and dementia: WHO guidelines.	"The management of depression in the form of antidepressants and/or psychological interventions should be provided"	21	37
8	Diabetes	Q16	Litke R, Garcharna LC, Jiwani S, Neugroschl J. Modifiable risk factors in Alzheimer disease and related dementias: a review. Clinical Therapeutics. 2021;43(6):953-965.	RR=1.9, CI 95% 1.2, 3.1	18	55

#	Risk/ protective factor	Question in checklist	Reference	Effect (extracted from the publication)	Average rating: %-rank effect size	Average rating: %-rank modify-ablity
			Dragioti E, Radua J, Solmi M, et al. Global population attributable fraction of potentially modifiable risk factors for mental disorders: a meta-umbrella systematic review. Mol Psychiatry. 2022;27(8):3510-3519.	RR=1.60, CI 95% 1.43, 1.79	36	55
			Yu JT, Xu W, Tan CC, et al. Evidence-based prevention of Alzheimer's disease: systematic review and meta-analysis of 243 observational prospective studies and 153 randomised controlled trials. J Neurol Neurosurg Psychiatry. 2020;91(11):1201-1209.	RR=1.69, CI 95% 1.51, 1.89	30	55
			Livingston, G., Huntley, J., Sommerlad, A., Ames, D., Ballard, C., Banerjee, S., ... & Mukadam, N. (2020). Dementia prevention, intervention, and care: 2020 report of the Lancet Commission. The Lancet, 396(10248), 413-446.	RR=1.5, CI 95% 1.3, 1.8	34	59
			World Health Organization. (2019). Risk reduction of cognitive decline and dementia: WHO guidelines.	"The management of diabetes in the form of medications and/or lifestyle interventions should be offered"	34	59
9	Green tea	Q4	Ran LS, Liu WH, Fang YY, et al. Alcohol, coffee and tea intake and the risk of cognitive deficits: a dose-response meta-analysis. Epidemiol Psychiatr Sci. 2021;30:e13.	RR=0.94, CI 95% 0.92, 0.97	98	4
10	Hearing impairment	Q11	Livingston, G., Huntley, J., Sommerlad, A., Ames, D., Ballard, C., Banerjee, S., ... & Mukadam, N. (2020). Dementia prevention, intervention, and care: 2020 report of the Lancet Commission. The Lancet, 396(10248), 413-446.	RR=1.9, CI 95% 1.4, 2.7	9	80
			Litke R, Garcharna LC, Jiwani S, Neugroschl J. Modifiable risk factors in Alzheimer disease and related dementias: a review. Clinical Therapeutics. 2021;43(6):953-965.	HR= 2.21, CI 95% 1.57, 3.12	8	65
11	Hypertension	Q19	Litke R, Garcharna LC, Jiwani S, Neugroschl J. Modifiable risk factors in Alzheimer disease and related dementias: a review. Clinical Therapeutics. 2021;43(6):953-965.	OR=4.8, CI 95% 2.0, 11.8	1	47
			de Heus RAA, Tzourio C, Lee EJL, et al. Association Between Blood Pressure Variability With Dementia and Cognitive Impairment: A Systematic Review and Meta-Analysis. Hypertension. 2021;78(5):1478-1489.	OR=1.16, CI 95% 1.04, 1.29	87	61
			Yu JT, Xu W, Tan CC, et al. Evidence-based prevention of Alzheimer's disease: systematic review and meta-analysis of 243 observational prospective studies and 153 randomised controlled trials. J Neurol Neurosurg Psychiatry. 2020;91(11):1201-1209.	RR=1.38, CI 95% 1.29, 1.47	55	61

#	Risk/ protective factor	Question in checklist	Reference	Effect (extracted from the publication)	Average rating: %-rank effect size	Average rating: %-rank modify-ablity
			de Heus RAA, Tzourio C, Lee EJL, et al. Association Between Blood Pressure Variability With Dementia and Cognitive Impairment: A Systematic Review and Meta-Analysis. Hypertension. 2021;78(5):1478-1489.	OR=1.12, CI 95% 1.02, 1.29	90	61
			Livingston, G., Huntley, J., Sommerlad, A., Ames, D., Ballard, C., Banerjee, S., ... & Mukadam, N. (2020). Dementia prevention, intervention, and care: 2020 report of the Lancet Commission. The Lancet, 396(10248), 413-446.	RR=1.96, CI 95% 1.2, 2.2	53	65
			Mentis AA, Dardiotis E, Efthymiou V, Chrousos GP. Non-genetic risk and protective factors and biomarkers for neurological disorders: a meta-umbrella systematic review of umbrella reviews. BMC Med. 2021;19(1):6.	VD: HR=1.59, CI 95% 1.20, 2.11; RR=0.64, CI 95% 0.42, 0.98	53	65
			World Health Organization. (2019). Risk reduction of cognitive decline and dementia: WHO guidelines.	"Management of hypertension may be offered"	53	65
			de Heus RAA, Tzourio C, Lee EJL, et al. Association Between Blood Pressure Variability With Dementia and Cognitive Impairment: A Systematic Review and Meta-Analysis. Hypertension. 2021;78(5):1478-1489.	OR=1.25, CI 95% 1.16, 1.35	69	65
12	Kidney disease	Q15	Kjaergaard AD, Ellervik C, Witte DR, Nordestgaard BG, Frikke-Schmidt R, Bojesen SE. Kidney function and risk of dementia: Observational study, meta-analysis, and two-sample mendelian randomization study. Eur J Epidemiol. 2022;37(12):1273-1284.	Moderately RR=1.91, CI 95% 1.21, 3.01	62	65
			Kjaergaard AD, Ellervik C, Witte DR, Nordestgaard BG, Frikke-Schmidt R, Bojesen SE. Kidney function and risk of dementia: Observational study, meta-analysis, and two-sample mendelian randomization study. Eur J Epidemiol. 2022;37(12):1273-1284.	Severely RR=1.91, CI 95% 1.21-3.01	16	83
	Management of chronic diseases	Q11-Q19	World Health Organization. (2019). Risk reduction of cognitive decline and dementia: WHO guidelines.		not reported	10
13	Mediterranean diet	Q3	Litke R, Garcharna LC, Jiwani S, Neugroschl J. Modifiable risk factors in Alzheimer disease and related dementias: a review. Clinical Therapeutics. 2021;43(6):953-965.	HR= 0.91, CI 95% 0.83, 0.98	93	10
			Bianchi VE, Herrera PF, Laura R. Effect of nutrition on neurodegenerative diseases. A systematic review. Nutr Neurosci. 2021;24(10):810-834.	overall number of studies	88	14
			Mentis AA, Dardiotis E, Efthymiou V, Chrousos GP. Non-genetic risk and protective factors and biomarkers for neurological disorders: a meta-umbrella systematic review of umbrella reviews. BMC Med. 2021;19(1):6.	e.g., RR=0.60, CI 95% 0.48, 0.77	88	14

#	Risk/ protective factor	Question in checklist	Reference	Effect (extracted from the publication)	Average rating: %-rank effect size	Average rating: %-rank modify-ablity
			García-Casares N, Gallego Fuentes P, Barbancho M, López-Gigosos R, García-Rodríguez A, Gutiérrez-Bedmar M. Alzheimer's Disease, Mild Cognitive Impairment and Mediterranean Diet. A Systematic Review and Dose-Response Meta-Analysis. J Clin Med. 2021;10(20).	RR=0.89, CI 95% 0.84, 0.93	94	14
14	Overweight	Q18	Litke R, Garcharna LC, Jiwani S, Neugroschl J. Modifiable risk factors in Alzheimer disease and related dementias: a review. Clinical Therapeutics. 2021;43(6):953-965.	RR=1.3, CI 95% 1.1, 1.6	64	17
			Livingston, G., Huntley, J., Sommerlad, A., Ames, D., Ballard, C., Banerjee, S., ... & Mukadam, N. (2020). Dementia prevention, intervention, and care: 2020 report of the Lancet Commission. The Lancet, 396(10248), 413-446.	RR=1.6, CI 95% 1.3, 1.9	88	27
			Tang X, Zhao W, Lu M, et al. Relationship between Central Obesity and the incidence of Cognitive Impairment and Dementia from Cohort Studies Involving 5,060,687 Participants. Neurosci Biobehav Rev. 2021;130:301-313.	HR=1.10, CI 95% 1.05, 1.15	88	27
			World Health Organization. (2019). Risk reduction of cognitive decline and dementia: WHO guidelines.	"Interventions for mid-life overweight and/or obesity may be offered"	88	27
15	Periodontal health	Q13	Asher S, Stephen R, Mäntylä P, Suominen AL, Solomon A. Periodontal health, cognitive decline, and dementia: A systematic review and meta-analysis of longitudinal studies. J Am Geriatr Soc. 2022;70(9):2695-2709.	HR=1.21, CI 95% 1.07, 1.38	78	40
16	Physical (in)activity	Q5	Yang X, Xu XY, Guo L, Zhang Y, Wang SS, Li Y. Effect of leisure activities on cognitive aging in older adults: A systematic review and meta-analysis. Front Psychol. 2022;13:1080740.	RR=0.83, CI 95% 0.74, 0.93	78	1
			Iso-Markku P, Kujala UM, Knittle K, Polet J, Vuoksimaa E, Waller K. Physical activity as a protective factor for dementia and Alzheimer's disease: systematic review, meta-analysis and quality assessment of cohort and case-control studies. Br J Sports Med. 2022;56(12):701-709.	RR=0.80, CI 95% 0.77, 0.84	75	1
			Livingston, G., Huntley, J., Sommerlad, A., Ames, D., Ballard, C., Banerjee, S., ... & Mukadam, N. (2020). Dementia prevention, intervention, and care: 2020 report of the Lancet Commission. The Lancet, 396(10248), 413-446.	RR=1.4, CI 95% 1.2, 1.7	65	1
			Mentis AA, Dardiotis E, Efthymiou V, Chrousos GP. Non-genetic risk and protective factors and biomarkers for neurological disorders: a meta-umbrella systematic review of umbrella reviews. BMC Med. 2021;19(1):6.	RR=0.76, CI 95% 0.66, 0.86; HR=0.62, CI 95% 0.52, 0.72	65	1

#	Risk/ protective factor	Question in checklist	Reference	Effect (extracted from the publication)	Average rating: %-rank effect size	Average rating: %-rank modify-ablity
			World Health Organization. (2019). Risk reduction of cognitive decline and dementia: WHO guidelines.	"Physical activity should be recommende d"	65	1
			Della Gatta F, Lacorte E, Fabrizi E, et al. Exploring the association of early life physical activity and risk of dementia: a systematic review. Minerva Med. 2021;112(4):448-455.	OR=0.63, CI 95% 0.39, 1.00	42	44
17	Playing musical instrument	Q2	Arafa A, Teramoto M, Maeda S, et al. Playing a musical instrument and the risk of dementia among older adults: a systematic review and meta-analysis of prospective cohort studies. BMC Neurol. 2022;22(1):395.	HR=0.64, CI 95% 0.41, 0.98	43	13
18	Post-traumatic stress disorder	Q17	Stafford J, Chung WT, Sommerlad A, Kirkbride JB, Howard R. Psychiatric disorders and risk of subsequent dementia: Systematic review and meta-analysis of longitudinal studies. Int J Geriatr Psychiatry. 2022;37(5).	HR=1.70, CI 95% 1.45, 2.00	33	65
19	Psychotic disorders	Q17	Miniawi SE, Orgeta V, Stafford J. Non-affective psychotic disorders and risk of dementia: a systematic review and meta-analysis. Psychol Med. 2022;52(15):1-13.	RR=2.52, CI 95% 1.67, 3.89	3	72
			Stafford J, Chung WT, Sommerlad A, Kirkbride JB, Howard R. Psychiatric disorders and risk of subsequent dementia: Systematic review and meta-analysis of longitudinal studies. Int J Geriatr Psychiatry. 2022;37(5).	RR=2.19, CI 95% 1.44, 3.31	11	95
20	Purpose in life	Q7	Sutin DAR, Luchetti M, Aschwanden D, Stephan Y, Sesker AA, Terracciano A. Sense of meaning and purpose in life and risk of incident dementia: New data and meta-analysis. Arch Gerontol Geriatr. 2023;105:104847.	HR=0.76, CI 95% 0.72, 0.79	62	17
			Sutin AR, Aschwanden D, Luchetti M, Stephan Y, Terracciano A. Sense of Purpose in Life Is Associated with Lower Risk of Incident Dementia: A Meta-Analysis. J Alzheimers Dis. 2021;83(1):249-258.	HR=0.77, CI 95% 0.73, 0.81	62	24
21	Smoking	Q9	Litke R, Garcharna LC, Jiwani S, Neugroschl J. Modifiable risk factors in Alzheimer disease and related dementias: a review. Clinical Therapeutics. 2021;43(6):953-965.	RR=2.3, CI 95% 1.3, 4.1	5	19
			Livingston, G., Huntley, J., Sommerlad, A., Ames, D., Ballard, C., Banerjee, S., ... & Mukadam, N. (2020). Dementia prevention, intervention, and care: 2020 report of the Lancet Commission. The Lancet, 396(10248), 413-446.	RR=1.6, CI 95% 1.2, 2.2	5	32
			Mentis AA, Dardiotis E, Efthymiou V, Chrousos GP. Non-genetic risk and protective factors and biomarkers for neurological disorders: a meta-umbrella systematic review of umbrella reviews. BMC Med. 2021;19(1):6.	RR=1.13, CI 95% 1.05, 1.22	5	32

#	Risk/ protective factor	Question in checklist	Reference	Effect (extracted from the publication)	Average rating: %-rank effect size	Average rating: %-rank modify-ablity
			World Health Organization. (2019). Risk reduction of cognitive decline and dementia: WHO guidelines.	"Interventions for tobacco cessation should be offered"	5	32
22	Social integration/ isolation	Q6	Qiao L, Wang G, Tang Z, et al. Association between loneliness and dementia risk: A systematic review and meta-analysis of cohort studies. Front Hum Neurosci. 2022;16:899814.	RR=1.72, CI 95% 1.32, 2.23	76	18
			Zhao YL, Qu Y, Ou YN, Zhang YR, Tan L, Yu JT. Environmental factors and risks of cognitive impairment and dementia: A systematic review and meta-analysis. Ageing Res Rev. 2021;72:101504.	HR=0.82, 95% CI 0.76, 0.90	79	18
			Dragioti E, Radua J, Solmi M, et al. Global population attributable fraction of potentially modifiable risk factors for mental disorders: a meta-umbrella systematic review. Mol Psychiatry. 2022;27(8):3510-3519.	RR=1.59, CI 95% 1.32, 1.85	38	18
			Livingston, G., Huntley, J., Sommerlad, A., Ames, D., Ballard, C., Banerjee, S., ... & Mukadam, N. (2020). Dementia prevention, intervention, and care: 2020 report of the Lancet Commission. The Lancet, 396(10248), 413-446.	RR=1.6, CI 95% 1.3, 1.9	41	18
			Litke R, Garcharna LC, Jiwani S, Neugroschl J. Modifiable risk factors in Alzheimer disease and related dementias: a review. Clinical Therapeutics. 2021;43(6):953-965.	OR=1.64, CI 95% 1.05, 2.56	34	18
			World Health Organization. (2019). Risk reduction of cognitive decline and dementia: WHO guidelines.	"Social participation and social support (…) should be supported"	41	18
23	Stress	Q10	Luo J, Beam CR, Gatz M. Is Stress an Overlooked Risk Factor for Dementia? A Systematic Review from a Lifespan Developmental Perspective. Prev Sci. 2022.	58% studies on early life stress, 57% on psychological stress in midlife, 42% on late life stress	97	37
			Yu JT, Xu W, Tan CC, et al. Evidence-based prevention of Alzheimer's disease: systematic review and meta-analysis of 243 observational prospective studies and 153 randomised controlled trials. J Neurol Neurosurg Psychiatry. 2020;91(11):1201-1209.	RR=1.65, CI 95% 1.19, 2.04	33	37
			Stuart KE, Padgett C. A Systematic Review of the Association Between Psychological Stress and Dementia Risk in Humans. J Alzheimers Dis. 2020;78(1):335-352.	most of the studies	not reported	65
24	Sleep disturbances	Q12	Shi, L., Chen, S. J., Ma, M. Y., Bao, Y. P., Han, Y., Wang, Y. M., ... & Lu, L. (2018). Sleep disturbances increase the risk of dementia: a system , stats(rho p)atic review	RR=1.53, CI 95% 1.07, 2.18	n/a	n/a

#	Risk/ protective factor	Question in checklist	Reference	Effect (extracted from the publication)	Average rating: %-rank effect size	Average rating: %-rank modify-ablity
			and meta-analysis. Sleep medicine reviews, 40, 4-16.			
**Excluded from checklist**						
1	Benzodiazepines		Ettcheto M, Olloquequi J, Sánchez-López E, et al. Benzodiazepines and Related Drugs as a Risk Factor in Alzheimer's Disease Dementia. Front Aging Neurosci. 2019;11:344.	RR=1.22, CI 95% 1.18, 1.25	48	40
			Dragioti E, Radua J, Solmi M, et al. Global population attributable fraction of potentially modifiable risk factors for mental disorders: a meta-umbrella systematic review. Mol Psychiatry. 2022;27(8):3510-3519.	RR=1.49, CI 95% 1.30, 1.72	77	40
			Ferreira P, Ferreira AR, Barreto B, Fernandes L. Is there a link between the use of benzodiazepines and related drugs and dementia? A systematic review of reviews. Eur Geriatr Med. 2022;13(1):19-32.	RR=1.22, CI 95% 1.18, 1.25	66	40
2	Education		Livingston, G., Huntley, J., Sommerlad, A., Ames, D., Ballard, C., Banerjee, S., ... & Mukadam, N. (2020). Dementia prevention, intervention, and care: 2020 report of the Lancet Commission. The Lancet, 396(10248), 413-446.	RR=1.6, CI 95% 1.3, 2.0	51	86
			Litke R, Garcharna LC, Jiwani S, Neugroschl J. Modifiable risk factors in Alzheimer disease and related dementias: a review. Clinical Therapeutics. 2021;43(6):953-965.	OR=0.42, CI 95% 0.37, 0.48	21	87
			Yu JT, Xu W, Tan CC, et al. Evidence-based prevention of Alzheimer's disease: systematic review and meta-analysis of 243 observational prospective studies and 153 randomised controlled trials. J Neurol Neurosurg Psychiatry. 2020;91(11):1201-1209.	RR=0.49, CI 95% 0.40, 0.62	27	87
			Maccora J, Peters R, Anstey KJ. What does (low) education mean in terms of dementia risk? A systematic review and meta-analysis highlighting inconsistency in measuring and operationalising education. SSM Popul Health. 2020;12:100654.	OR=1.45, CI 95% 1.29, 1.63	50	87
3	Environmental Atmosphere		Oliveira M, Padrão A, Ramalho A, et al. Geospatial Analysis of Environmental Atmospheric Risk Factors in Neurodegenerative Diseases: A Systematic Review. Int J Environ Res Public Health. 2020;17(22).	„was related to ementia“	n/a	74
4	B Vitamins		Zhou J, Sun Y, Ji M, Li X, Wang Z. Association of Vitamin B Status with Risk of Dementia in Cohort Studies: A Systematic Review and Meta-Analysis. J Am Med Dir Assoc. 2022;23(11):1826.e1821-1826.e1835.	HR=1.57, CI 95% 1.01, 2.46	42	12
5	Anticholinergic medication		Litke R, Garcharna LC, Jiwani S, Neugroschl J. Modifiable risk factors in Alzheimer disease and related dementias: a review. Clinical Therapeutics. 2021;43(6):953-965.	OR=1.49, 95% CI 1.44, 1.54	96	40

#	Risk/ protective factor	Question in checklist	Reference	Effect (extracted from the publication)	Average rating: %-rank effect size	Average rating: %-rank modify-ablity
6	Cerebral microbleeds		Hussein AS, Shawqi M, Bahbah EI, et al. Do cerebral microbleeds increase the risk of dementia? A systematic review and meta-analysis. IBRO Neurosci Rep. 2023;14:86-94.	RR=1.84, CI 95% 1.25, 4.26	22	92
7	Coronary artery bypass grafting		Yu JT, Xu W, Tan CC, et al. Evidence-based prevention of Alzheimer's disease: systematic review and meta-analysis of 243 observational prospective studies and 153 randomised controlled trials. J Neurol Neurosurg Psychiatry. 2020;91(11):1201-1209.	RR= 1.71, CI 95% 1.04, 2.79	28	92
8	Frailty		Guo CY, Sun Z, Tan CC, Tan L, Xu W. Multi-Concept Frailty Predicts the Late-Life Occurrence of Cognitive Decline or Dementia: An Updated Systematic Review and Meta-Analysis of Longitudinal Studies. Front Aging Neurosci. 2022;14:855553.	RR=1.37, CI 95% 1.13, 1.66	41	66
**Not among top 50**						
1	Air pollution		Zhao YL, Qu Y, Ou YN, Zhang YR, Tan L, Yu JT. Environmental factors and risks of cognitive impairment and dementia: A systematic review and meta-analysis. Ageing Res Rev. 2021;72:101504.	PM2.5: HR=1.24, CI 95% 1.17, 1.31	74	75
			Zhao YL, Qu Y, Ou YN, Zhang YR, Tan L, Yu JT. Environmental factors and risks of cognitive impairment and dementia: A systematic review and meta-analysis. Ageing Res Rev. 2021;72:101504.	NO2: HR=1.07, CI 95% 1.02, 1.12	97	75
			Chandra M, Rai CB, Kumari N, et al. Air Pollution and Cognitive Impairment across the Life Course in Humans: A Systematic Review with Specific Focus on Income Level of Study Area. Int J Environ Res Public Health. 2022;19(3).	PM2.5: HR=1.04, CI 95% 1.03, 1.05	75	75
			Livingston, G., Huntley, J., Sommerlad, A., Ames, D., Ballard, C., Banerjee, S., ... & Mukadam, N. (2020). Dementia prevention, intervention, and care: 2020 report of the Lancet Commission. The Lancet, 396(10248), 413-446.	RR=1.1, CI 95% 1.1, 1.1	88	76
2	Aluminium pollution		Zhao YL, Qu Y, Ou YN, Zhang YR, Tan L, Yu JT. Environmental factors and risks of cognitive impairment and dementia: A systematic review and meta-analysis. Ageing Res Rev. 2021;72:101504.	OR=1.35, CI 95% 1.14, 1.59	61	74
3	Blood pressure variability		Jia P, Lee HWY, Chan JYC, Yiu KKL, Tsoi KKF. Long-Term Blood Pressure Variability Increases Risks of Dementia and Cognitive Decline: A Meta-Analysis of Longitudinal Studies. Hypertension. 2021;78(4):996-1004.	HR=1.11, CI 95% 1.05, 1.17	85	73
4	Chronic noise exposure		Meng L, Zhang Y, Zhang S, et al. Chronic Noise Exposure and Risk of Dementia: A Systematic Review and Dose-Response Meta-Analysis. Front Public Health. 2022;10:832881.	RR=1.16, CI 95% 1.12, 1.20	65	62
5	Personality (neuroticism)		Aschwanden D, Strickhouser JE, Luchetti M, Stephan Y, Sutin AR, Terracciano A. Is personality associated with dementia risk?	HR=1.24, CI 95% 1.17, 1.31	73	86

#	Risk/ protective factor	Question in checklist	Reference	Effect (extracted from the publication)	Average rating: %-rank effect size	Average rating: %-rank modify-ablity
			A meta-analytic investigation. Ageing Research Reviews. 2021;67:101269.			
6	Solvents		Zhao YL, Qu Y, Ou YN, Zhang YR, Tan L, Yu JT. Environmental factors and risks of cognitive impairment and dementia: A systematic review and meta-analysis. Ageing Res Rev. 2021;72:101504.	OR=1.14, CI 95% 1.07, 1.22	89	84
7	Traumatic brain injury		Yu JT, Xu W, Tan CC, et al. Evidence-based prevention of Alzheimer's disease: systematic review and meta-analysis of 243 observational prospective studies and 153 randomised controlled trials. J Neurol Neurosurg Psychiatry. 2020;91(11):1201-1209.	RR=1.35, CI 95% 1.18, 1.54	60	89
			Zhang J, Zhang Y, Zou J, Cao F. A meta-analysis of cohort studies: Traumatic brain injury and risk of Alzheimer's Disease. PLoS One. 2021;16(6):e0253206.	RR=1.17, CI 95% 1.05, 1.29	86	89
			Litke R, Garcharna LC, Jiwani S, Neugroschl J. Modifiable risk factors in Alzheimer disease and related dementias: a review. Clinical Therapeutics. 2021;43(6):953-965.	HR=1.2, CI 95% 1.2, 1.3	81	89
			Livingston, G., Huntley, J., Sommerlad, A., Ames, D., Ballard, C., Banerjee, S., ... & Mukadam, N. (2020). Dementia prevention, intervention, and care: 2020 report of the Lancet Commission. The Lancet, 396(10248), 413-446.	RR=1.8, CI 95% 1.5, 2.2	77	91

Notes: CI, confidence interval; HR, hazard ratio; MMSE, mini mental state examination; NO2, Nitrogen Dioxide; OR, odds ratio; PM2.5, Particulate Matter with diameters <=2.5 micrometers; SMD, standardized mean difference; RR, risk ratio; VD, vascular dementia.
