# Supplementary material for: Practicality of a patient self-assessment checklist to manage dementia risk factors in GP practices
Source: Sci Rep. 2025 May 16;15:17064. doi: 10.1038/s41598-025-01455-8 (PMC12084373; doi:10.1038/s41598-025-01455-8)
Supplement: Supplementary file 2 — Supplementary Material 2 [file 41598_2025_1455_MOESM2_ESM.pdf]

Supplementary File 2

To the manuscript 'Practicality of a patient self-assessment checklist to manage dementia risk factors in GP practices' (Rodriguez et al.)

General practitioners (GP) experience questionnaire with the respective results (English translation)

Personal Information

1. What gender do you assign yourself to?
   71.4% Female, 28.6% Male, 0.0% Non-binary
2. How old are you?
   M = 44.8 years (*SD* = 7.8 years)
3. How long have you been working in your profession?
   21.4 % 0-10 years, 57.1 % 10-20 years, 21.4 % more than 20 years

Use of the Risk Factor Checklist

4. How many patients have you distributed the Risk Factor Checklist to?
   M = 5.9 patients (*SD* = 3.3 patients)
5. How many patients have refused to complete the Risk Factor Checklist?
   None: 69.2 %
   1.5 % of patients: 7.7 %
   2 % of patients: 7.7 %
   20 % of patients: 7.7 %
   25% of patients: 7.7 %
6. What percentage of the Risk Factor Checklist did your patients complete?
   90 % of the checklist: 20.0 %
   100 % of the checklist: 80.0 %
   M = 98.0 % of the checklist (*SD* = 4.2 % of the checklist)
7. Which Risk Factor Checklist questions were most often omitted?
   alcohol, renal dysfunction, playing a musical instrument and drinking alcoholic beverages, medical questions on health status, stress, none (n=4)
8. Where was the Risk Factor Checklist completed by most patients? (multiple answer choices)
   50.0 % in the waiting room, 14.3 % at home, 64.3% in a personal conversation with the patient, 14.3% Other: in the lab with a nurse, personal conversation with nurse
9. Did patients have sufficient time to complete the Risk Factor Checklist during their visit? 100.0 % Yes, 0.0% No
10. For which target groups did you find the use of the Risk Factor Checklist important?
    - those older than 60 years
    - those older than 60 with chronic conditions
    - those older than 70 years (n=2)
    - those older than 75 years
    - score for younger people important to show them risk factors so that there is still time to change them
    - older patients who live alone with psychiatric indication
    - patients with insecurities and memory problems and newly retired
    - patients scared of developing dementia or who have dementia in their family/ patients with mild cognitive impairment
    - patients with mild cognitive impairment
    - older patients with signs of cognitive deficits
    - those with dementia
11. For which target groups did the use of the Risk Factor Checklist not make sense?
    - none (n=3)
    - if German is not their mother tongue and those with severely reduced intelligence
    - patients older than 90 years
    - old people and with dementia
    - patients with dementia (4x)
    - patients with dementia and in a nursing home
    - strongly depressed and those with dementia
12. What problems came up while using the Risk Factor Checklist?
    28.6 % No: none (n=3), no problems the questions were honestly answered
    14.3 % Time intensive: time in the office, some patients had to take time to think about some questions
    21.4 % Reading: too long phrases on the checklist, patients had to read out loud some of the questions repeatedly to understand them, difficulties reading the writing but less due to language issues
    35.7 % Misunderstanding questions: some questions are very subjective or general like cognitive demanding leisure activities, questions with less than and Yes/No were problematic for those patients asked questions, sometimes the

negative questions like less than 1 cup were misunderstood, questions were misunderstood, I had the impression that one or two questions were misunderstood and answered incorrectly accordingly

13. Have you been able to use the Risk Factor Checklist to gain important information about your patients that you would not have known otherwise?
    71.4 % Yes, 28.6 % No
14. Which of the risk factors on the Risk Factor Checklist have proven most important to you? purpose in life as indication for possible suicide that requires actions, no musical instrument and demanding leisure activities and sleep problems, living situation, social contacts and leisure activities and sleep problems, social contact and cognitive activation, social contact and cognitive activation and hearing, social contacts and alcohol, physical exercise and nutrition, depression (n=2)
15. Based on the indications of the Risk Factor Checklist, what actions have you taken?
    69.2 % Personal lifestyle advice (*M* = 65.0 %, *SD* = 29.3 % of patients)
    7.7 % Referral to programs of health insurance companies (*M* = 0.0 % of patients)
    38.5 % Referral to a specialist or therapist (*M* = 62.5 %, *SD* = 45.0 % of patients)
    30.8 % Prescription of medication (*M* = 3.3 %, *SD* = 5.8 % of patients)
    15.4 % Initiation of prevention or rehabilitation measures (*M* = 26.5 %, *SD* = 9.2 % of patients)
    61.5 % Refer to guides/brochures or courses (*M* = 55.8 %, *SD* = 38.4 % of patients)
    7.7 % Other: explain repetitively the need for prevention and demand compliance (*M* = 30.0 %, *SD* = 0.0 % of patients)
16. Do you feel that the Risk Factor Checklist motivated patients to modify existing risk factors?
    21.4 % Yes, 50.0 % Partly, 28.6 % No

Treatment Context

17. What impact did the Risk Factor Checklist have on your personal workload?
    28.6 % No: none (n=3), those questionnaires did not burden me
    35.7 % Moderately: little, moderately increased (n =2), a bit time intensive due to having to explain some questions, according to my nurses it takes time in the waiting room that is no problem if done sporadically
    21.4 % Very: time intensive, more work as patients want to know why they have to fill it out and has to be evaluated and discussed, time required that would usually take an entire geriatric assessment to complete in
18. Apart from this Risk Factor Checklist, do you already use checklists in your practice?
    57.1 % No
    42.9 %Yes, these are: Bathel index and several questionnaires, DemTec, MMSE, Urology, GDS, MMSE, DemTec, Wells score, geriatric assessments, center score, CHA2DS2 Vasc Score, WAS-BLED-Score
19. Were you already familiar with modifiable risk factors for dementia before using the checklist?
    35.7 % Yes, 35.7 % Partly, 28.6 % No
20. Would you like more information on the topic of "Risk factors for dementia"?
    25.0 % Yes in an online event, 66.7 % Yes in form of a brochure, 8.3 % No
21. Do you have a method that allows you to monitor the treatment of chronic diseases (dementia-specific risk factors) in the long term?
    35.7 % Yes, this: regular contact to patients, control after 6 months, optimizing medication, 3-year check-ups to discuss physical activity, alcohol consumption, hearing and vision abilities, lab results, and obesity
    64.3 % No: How should such a method be designed? Time-saving (n=2), questionnaires, re-call-system, DMP, GBA, DemTect, MMSE, digital tool similar to ARRIBA, simple digital that can be used by assistants
22. Based on the information you gained about your patients from the Risk Factor Checklist, what would you need urgently to be able to provide adequate care for your patients?
    30.8 % Prevention/ rehabilitation measures. Examples: dementia/ brain aging
    69.2 % Brochures/guides to hand out. Examples: healthy nutrition, dementia, dementia and how to deal with it
    38.5 % Offers from health insurance companies. Examples: activities and health lifestyle for staying fit in old age, dealing with dementia for family caregivers
    7.7 % Courses. Examples: playing a musical instrument and healthy nutrition
    7.7 % Other. Examples: evidence-based therapies

Need for improvement

23. Would you prefer a digital version of the Risk Factor Checklist?
    75.0 % No
    25.0 % Yes: What should be considered for a digital version? Age-appropriately easy and communicative for the patient to complete on his/her own, integrated in existing patient management system, creation of a program (Tork, Smileys) for better communication with patients
24. Are there any risk factors missing from the Risk Factor Checklist that you feel are important?
    76.9 % No, 23.1 % Yes, the following: body hygiene, medications that increase dementia risk (could be completed by medical assistants), what people wish for in their life
25. Would you add a question about the genetic predisposition to be filled out by the doctor?
    23.1 % Yes, 76.9 % No
26. Is the evaluation of the Risk Factor Checklist sufficient in its current state?
    83.3 % Yes
    16.7 % No: What should be changed (e.g. risk score, graphic/visual representation)? Weighing of the risk factors (e.g., score) that indicates at which cut-off a conversation has to take place, graphic/visual illustration, (scoring criteria are currently not available)
27. The KogFit Risk Factor Checklist is useful and helpful.

Disagree: 21.4 %
Neutral: 21.4 %
Agree: 42.9 %
Strongly agree: 14.3 %
M = 3.5, *SD* = 1.0 (Likert: strongly disagree [1] to strongly agree [5])

28. The language of the KogFit risk factor checklist should be simplified.
M = 3.6, *SD* = 1.3 (Likert: strongly disagree [1] to strongly agree [5])

29. The KogFit Risk Factor Checklist should include more visual aids (e.g. symbols, etc.).
M = 3.5, *SD* = 1.3 (Likert: strongly disagree [1] to strongly agree [5])

30. I will be using the KogFit Risk Factor Checklist in my practice.
Strongly Disagree: 7.1 %
Disagree: 21.4 %
Neutral: 42.9%
Agree: 21.4 %
Strongly Agree: 7.1 %
M = 3.0, *SD* = 1.0 (Likert: strongly disagree [1] to strongly agree [5])

31. I don't see any need for the KogFit Risk Factor Checklist.
M = 2.0, *SD* = 1.3 (Likert: strongly disagree [1] to strongly agree [5])

32. The KogFit Risk Factor Checklist takes up too much time.
M = 2.6, *SD* = 1.4 (Likert: strongly disagree [1] to strongly agree [5])

33. Non-modifiable factors should also be included in the check-list.
M = 2.5, *SD* = 1.1 (Likert: strongly disagree [1] to strongly agree [5])

34. Do you want to make further comments?
- the checklist should be conceptualized so that it can easily be used by the assistant with the patient and then in consultation with the GP
- for the question with “less than” they had to think and yes and no were easily mixed-up so that translation in the correct color was not correct
- checklist can only be used with those in which the disease has not progressed too much and can fill it out only with help of the nurses
- the questions in the checklist are usually known to the GP who makes the diagnosis and the important questions for the lifestyle
- something like this is not going to work in a GPs office not enough time is not being paid
- I am interested in further versions of the checklist and results from this evaluation
