# Supplementary material for: Practicality of a patient self-assessment checklist to manage dementia risk factors in GP practices
Source: Sci Rep. 2025 May 16;15:17064. doi: 10.1038/s41598-025-01455-8 (PMC12084373; doi:10.1038/s41598-025-01455-8)
Supplement: Supplementary file 3 — Supplementary Material 3 [file 41598_2025_1455_MOESM3_ESM.pdf]

Supplementary File 3

To the manuscript ‘Practicality of a patient self-assessment checklist to manage dementia risk factors in GP practices’ (Rodriguez et al.)

Modified risk factor checklist based on the feedback of general practitioners (English translation).

Lifestyle

Are your leisure or work activities mentally demanding?	☐ No	☐ Yes
Do you play a musical instrument?	☐ No	☐ Yes
Do you eat **a lot of** fruits/ vegetables/ nuts/ fish and **little** meat?	☐ No	☐ Yes
Do you drink **more** than 1 cup of coffee or green tea a day?	☐ No	☐ Yes
Do you do **intense physical activity** at least 2x/week?<br>(e.g. sports, strenuous gardening)	☐ No	☐ Yes
Do you have **enough** social contacts?	☐ No	☐ Yes
Do you see a purpose in your life?	☐ No	☐ Yes

Do you drink **2 or more** glasses of alcoholic beverages a day?<br>(e.g. beer, schnapps, etc.)	☐ No	☐ Yes
Are you a smoker? (does not include e-cigarettes/cigars or occasional smokers)	☐ No	☐ Yes
Do you have **a lot of** stress?	☐ No	☐ Yes

Health state *(If you are unsure, you can also ask your doctor)*

Do you have hearing impairment? (e.g. diagnosis or others say you hear badly)	☐ No	☐ Yes
Do you have trouble falling/ staying asleep?	☐ No	☐ Yes
Do you have peridontitis? (e.g. gingivitis, bleeding gums, periodontitis, loose teeth)	☐ No	☐ Yes
Do you have chronic pain?	☐ No	☐ Yes
Do you have renal impairment?	☐ No	☐ Yes
Do you have diabetes?	☐ No	☐ Yes
Do you have a mental illness? (e.g. depression, anxiety disorder, post-traumatic stress disorder, psychotic illness)	☐ No	☐ Yes
Are you strongly overweight?	☐ No	☐ Yes
Do you have high blood pressure ($\geq$140/90 mm Hg) and do **not** take any medication for it?	☐ No	☐ Yes

Evaluation
This checklist contains risk factors that are scientifically known to be associated with a higher risk of cognitive impairment in older age. Your crosses indicate what risk factors apply to you. For each question, take a look at where their cross is.
Cross in the green area: Very good. Your behavior and your health state are beneficial.
Cross in the red area: There is a risk factor. Look at the question again: Is there anything you can do about this situation? The more crosses you have in red areas, the more important it is for you to reflect on your current situation and take actions on improving it. Talk to a person you trust about possibilities and offers.
